# Splicing therapy for neuromuscular disease^☆^

**DOI:** 10.1016/j.mcn.2013.04.005

**Published:** 2013-09

**Authors:** Andrew G.L. Douglas, Matthew J.A. Wood

**Affiliations:** Department of Physiology, Anatomy and Genetics, University of Oxford, UK

**Keywords:** 2′OMePS, 2′-O-methyl phosphorothioate, 2′MOE-PS, 2′-O-methoxyethyl phosphorothioate, AON, antisense oligonucleotide, CPP, cell-penetrating peptide, DMD, Duchenne muscular dystrophy, PMO, phosphorodiamidate morpholino, PPMO, peptide-conjugated phosphorodiamidate morpholino, SMA, spinal muscular atrophy, DMD, SMA, Antisense, Splicing, Exon skipping, Exon inclusion

## Abstract

Duchenne muscular dystrophy (DMD) and spinal muscular atrophy (SMA) are two of the most common inherited neuromuscular diseases in humans. Both conditions are fatal and no clinically available treatments are able to significantly alter disease course in either case. However, by manipulation of pre-mRNA splicing using antisense oligonucleotides, defective transcripts from the *DMD* gene and from the *SMN2* gene in SMA can be modified to once again produce protein and restore function. A large number of *in vitro* and *in vivo* studies have validated the applicability of this approach and an increasing number of preliminary clinical trials have either been completed or are under way. Several different oligonucleotide chemistries can be used for this purpose and various strategies are being developed to facilitate increased delivery efficiency and prolonged therapeutic effect. As these novel therapeutic compounds start to enter the clinical arena, attention must also be drawn to the question of how best to facilitate the clinical development of such personalised genetic therapies and how best to implement their provision.

## Introduction

Inherited neurological disorders have long suffered from a relative paucity of effective treatment options. Whilst our knowledge of supportive care along with symptomatic and palliative treatments has improved considerably over recent decades, the same has not been true of therapies aimed at the molecular defects themselves, despite many of the responsible genes and pathological mechanisms being known. This frustrating situation, however, is starting to change. We now know enough about the molecular pathogenesis of an increasing number of the monogenic neurological disorders to be able to design targeted disease-modifying genetic therapies for the first time. One such disease for which the development of targeted therapy is already far advanced is Duchenne muscular dystrophy (DMD). Another disease where such treatment is currently under rapid development is spinal muscular atrophy (SMA). This review will explain how the manipulation of RNA splicing can be used as an effective corrective therapy for these two classic genetic conditions. We outline the molecular pathogenesis and splicing biology of DMD and SMA, explain the design and use of antisense oligonucleotides in therapeutic exon skipping and exon inclusion respectively, and discuss the delivery of oligonucleotide drugs to muscle, heart and the central nervous system. We conclude with some thoughts on the future of splicing therapies in clinical practice.

## Duchenne muscular dystrophy

DMD is a genetic disease of the muscle caused by mutations in the *DMD* gene, which lies at chromosomal locus Xp21 (Emery, 2002). The condition affects around 1 in 3500 live male births and generally presents in early childhood with proximal muscle weakness. Affected boys may present with gross motor delay and there can also be a non-progressive cognitive impairment of variable degree in around one third of cases. The usual natural history is one of gradually progressive weakness so that ambulation is lost by the teenage years. Histologically there is replacement of skeletal muscle tissue with fibrofatty infiltration (Zhou and Lu, 2010). This can result in a rubbery pseudohypertrophy of the calf muscles, which is a characteristic feature of the condition. The depleted muscle fibres show evidence of dystrophy, with repeating cycles of necrosis, regeneration and fibrosis resulting in unequal fibre size. The dystrophic process gradually affects the diaphragm and other respiratory muscles, eventually leading to respiratory failure, and cardiac muscle is also affected, resulting in a dilated cardiomyopathy (Fayssoil et al., 2010). Cardiorespiratory failure is the primary cause of mortality in such patients and death typically occurs in early adulthood. Current treatment options are limited, with supportive care and corticosteroid treatment being the mainstays of conventional therapy (Bushby et al., 2010a, 2010b; Moxley et al., 2010). Although advances in such care have delivered considerable improvements in patient survival over recent decades, there remains a pressing need for disease-modifying therapy (Eagle et al., 2002).

### Molecular pathogenesis of dystrophinopathies

**Fig. 1:**
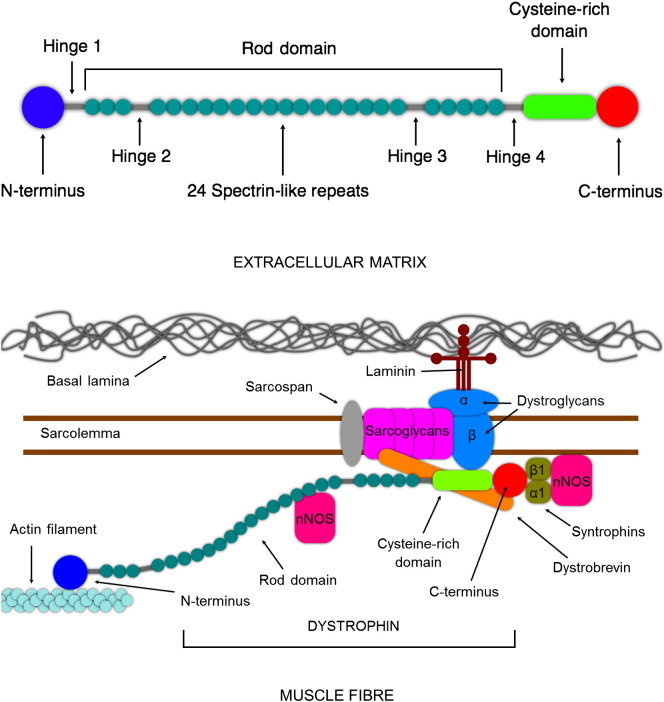
Dystrophin and the dystrophin-associated glycoprotein complex (DAGC). Top: features of the dystrophin protein. The N- and C-terminal regions contain functionally important binding sites whilst the rod domain acts as a linker. The central rod domain comprises 24 spectrin-like repeats interspersed by 4 hinge regions that are thought to help provide molecular flexibility. In addition, the rod domain contains further binding sites for actin and nNOS. Bottom: dystrophin connects the cytoskeleton to the sarcolemma *via* components of the DAGC, a large multiprotein complex which includes laminin, sarcoglycans, α- and β-dystroglycan, sarcospan, dystrobrevin and α1- and β1-syntrophin, as well as associated proteins such as NOS. The N-terminus binds to F-actin, whilst at the other end the cysteine-rich region binds β-dystroglycan and the C-terminus binds to syntrophins and dystrobrevin. The DAGC protein complex straddles the sarcolemma and binds *via* laminin to the basal lamina of the extracellular matrix.

The *DMD* gene encodes the protein dystrophin (Hoffman et al., 1987). At least seven major isoforms of differing lengths are encoded by this gene, each using an alternative intragenic promoter (Muntoni et al., 2003). The true number of isoforms is likely to be considerably higher, owing to the presence of multiple alternative splicing events. However, the full-length skeletal muscle isoform is a 427 kDa protein 3685 amino acids in length that localises to the sarcolemma (Zubrzycka-Gaarn et al., 1988). Here it plays a structural role, linking the cytoskeleton to the cell membrane and, *via* the dystrophin-associated glycoprotein complex (DAGC), beyond to the extracellular matrix. This connective function allows for the transmission of force from the contractile cytoskeletal elements of skeletal myofibres to extracellular structures. It is also important for maintaining the integrity of the muscle cell membrane (Davies and Nowak, 2006). The structure of full-length dystrophin allows it to carry out this role (see Fig. 1). On a simplistic level, the protein can be thought of as something akin to a bungee rope in that its central portion consists of a long, repetitive “rope-like” region (called the rod domain), whilst at either end there are molecular “hooks” to allow binding to cytoskeletal F-actin at one end (the N-terminus) and to the sarcolemmal DAGC at the other (the C-terminus). The rod domain is a coiled-coil region made of 24 spectrin-like repeats interspersed by 4 hinge regions (Ervasti, 2007). Although the rod domain is generally believed to have a large degree of functional redundancy in terms of dystrophin’s mechanical role, it does contain a further actin-binding domain and is also thought to interact with membrane phospholipids, nNOS and other cytoskeletal elements such as plectin, intermediate filaments and microtubules (Le Rumeur et al., 2010). The major actin-binding interaction at the N-terminus is mediated by two calponin homology domains. At the other end of the protein, just proximal to the C-terminus, a strong interaction takes place with the β-dystroglycan component of the DAGC *via* a cysteine-rich domain. The C-terminus itself binds other DAGC components such as syntrophins and α-dystrobrevin.

With this model in mind, it can easily be appreciated that a mutated dystrophin protein lacking either of its terminal ends would be functionally incapable. Indeed DMD patients usually have mutations that cause loss of the C-terminal domain, resulting in non-functional dystrophin. However, if a mutation were to lack only part of the central rod domain, the resulting protein could potentially still function tolerably well. This is borne out by the existence of the much milder condition Becker muscular dystrophy (BMD), which is allelic to DMD (Kingston et al., 1983). In BMD, patients typically express a truncated dystrophin protein that lacks a portion of the rod domain. Symptoms usually do not occur until late childhood, adolescence or adulthood and are generally a lot milder than DMD, with patients often maintaining ambulation well into middle and older age (Bushby and Garner-Medwin, 1993). As will be explained later, the presence of this milder phenotypic form of the disease provides the basis for how DMD can be treated by splice modulation.

The 2.4 Mb long *DMD* gene, located at cytogenetic locus Xp21.2–p21.1, has the distinction of being the longest known gene in the human genome (Boyce et al., 1991; den Dunnen et al., 1989). Seventy-nine exons are encoded by the full-length transcript (Roberts et al., 1993). However, despite its expansive genomic length, the fully spliced mature mRNA of full-length dystrophin is only some 14 kb long (Koenig et al., 1987). The implication of this is that whilst each exon is roughly of the order of 150 bp long (excluding the 2.3 kb final exon), they are separated by much longer introns that on average are about 30 kb long (although their individual lengths range from 107 bp for intron 14 up to over 319 kb for intron 1 of the brain full-length isoform). This feature of the *DMD* locus is likely to partly explain why single or multiple whole exon deletions are the most commonly found mutations in affected patients.

**Fig. 2:**
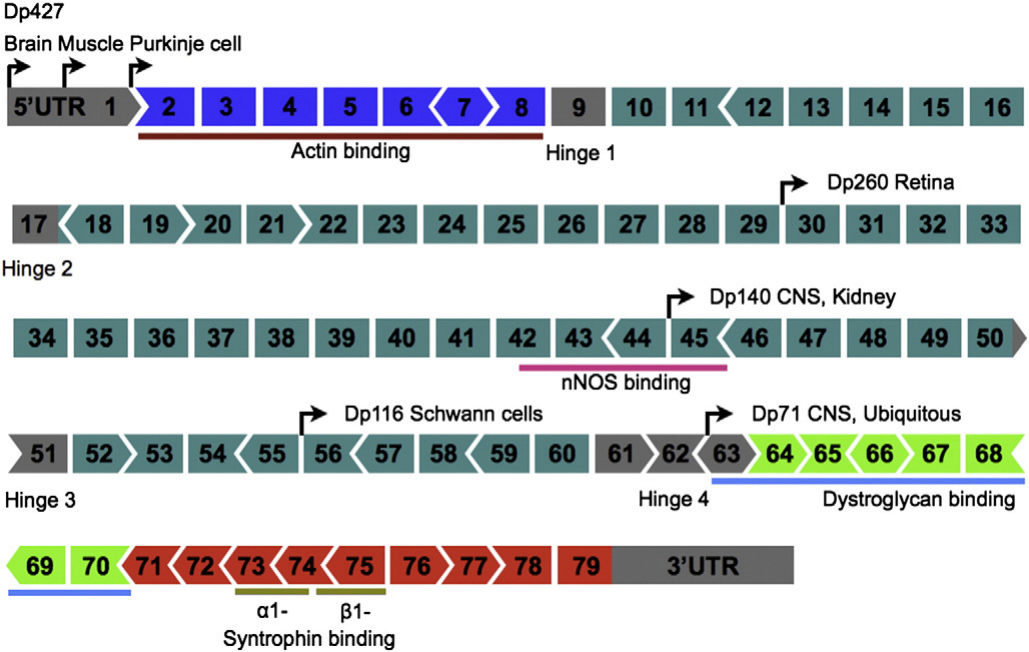
Structure of the *DMD* gene, including positions of promoters for different isoforms. The full-length dystrophin protein (Dp427) has three separate tissue-specific promoters, predominantly expressed in brain (also known as the cortical promoter), skeletal muscle and cerebellar Purkinje cells respectively. The Dp260 isoform is mainly expressed in retina and originates from a promoter within intron 29. Dp140 has a promoter in intron 44 and is present in the CNS and kidney, whilst Dp116 has an intron 55 promoter and is predominantly found in Schwann cells. Dp71 is expressed from an intron 62 promoter and is ubiquitously expressed, although it appears to play an especially important role within the CNS. Also shown are the locations of binding sites for actin, nNOS, dystroglycans and syntrophins. The diagram also shows how the 79 exons fit together in terms of the normal open reading frame. Each individual exon may coincide with positions 1, 2 or 3 of a codon in the normal open reading frame. This is represented by three alternative shapes at the ends of the exons. If the exons fit together, the reading frame is maintained. If an exon (or a block of exons) with differently shaped ends is deleted from the gene, the reading frame is disrupted and the result is DMD. However, deletion of an exon with ends of the same configuration will not affect the reading frame since the remaining exons will still fit together (the equivalent of BMD). Exons are coloured according to the domain they encode: N-terminus (blue), rod domain (dark green with hinge regions in grey), cysteine-rich domain (light green) and C-terminus (red).

Since exon–exon junctions do not always fall neatly at the ends of triplet base codons, deletions of certain exons can cause a shift of the open reading frame at the site of the new exon–exon junction in the spliced transcript (see Fig. 2). Such frameshifts invariably lead to the incorporation of a premature STOP codon in the near downstream region of the transcript. If such a dystrophin protein were to be made, it would be nonfunctional and possibly unstable and thus these out-of-frame mutations cause DMD. In addition, the presence of a premature termination codon in the middle of a transcript is recognised by the cell, which activates the nonsense-mediated mRNA decay pathway, degrading the transcript and curtailing production of aberrant protein (Buvoli et al., 2007). In contrast to this, if exon deletions occur where the normal open reading frame is maintained, functional dystrophin is usually still produced, resulting in the milder BMD phenotype (Monaco et al., 1988).

### Splicing biology of dystrophin

The extreme length of the *DMD* locus means that the RNA polymerase II enzyme takes around 16 hours to generate a single complete transcript (Tennyson et al., 1995). If a cell were to require the completion of the gene's transcription prior to splicing it, the whole process would be impossibly unwieldy. By necessity then, the cell commences pre-mRNA splicing concurrently with transcription. This co-transcriptional property appears to be a common feature of splicing in general and this impacts upon choice of available splice sites and on alternative exon selection (Kornblihtt et al., 2004). One interesting consequence of this prolonged time period needed for transcription is that rapidly dividing cells are unable to express dystrophin to any significant degree. The average time between cell divisions for cultured human myoblasts is in fact also around 16–17 h and so it may be that the paucity of dystrophin expression in such cells could in part be linked to this transcriptional time limitation (Blau et al., 1985; Nudel et al., 1988). Indeed, such a large gene is likely to have highly complex splicing and much remains uncertain regarding the precise order of splicing events. It may be, for example, that shorter introns are spliced out more quickly than longer ones. This would lead to a nonconsecutive exon splicing order, which would have clear implications for splice-directed therapies (Aartsma-Rus et al., 2006). Some of the studies into DMD multi-exon splicing (discussed below) lend support to this hypothesis.

**Fig. 3:**
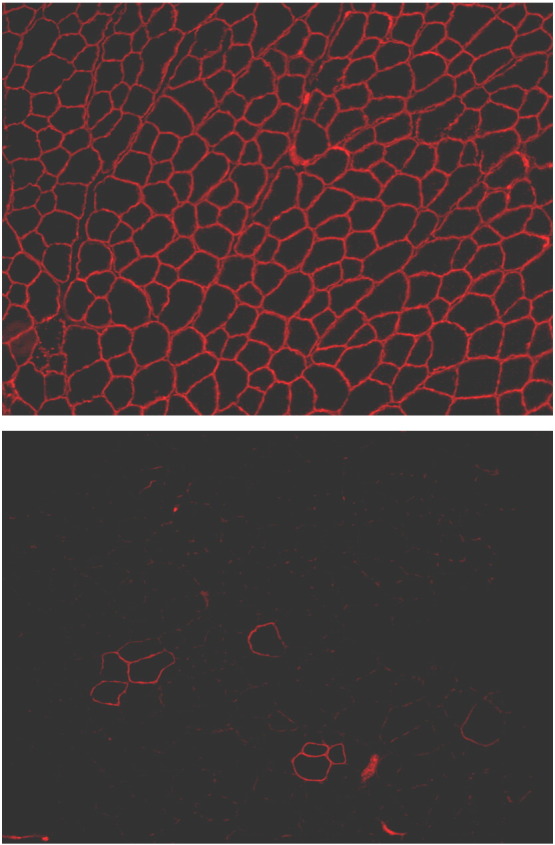
Immunohistochemical fluorescent staining of dystrophin in skeletal muscle fibres (tibialis anterior) in mice. Top: sarcolemmal localisation of dystrophin in a wild-type (C57 BL/10) mouse. Transverse section of muscle fibres shows generally uniformly sized angulated fbres with consistent dystrophin staining around the cell membrane Bottom: lack of dystrophin expression in the *mdx* mouse DMD model. The majority of fibres show no dystrophin expression. However, note the presence of occasional revertant fibres in *mdx* leading to small clusters of dystrophin positive fibres, the result of sporadic naturally occurring exon-skipping events that restore the reading frame.

Immunohistochemical staining for dystrophin in the muscle tissue sections of DMD patients shows an absence of the protein from the muscle sarcolemmal membrane. However, occasional isolated fibres can be found that still appear to express correctly localised dystrophin (see Fig. 3) (Arechavala-Gomeza et al., 2010). These fibres, known as revertant fibres, are thought to be examples of where, by chance, second superadded mutations or intrinsic aberrant splicing events have led to the missing out or “skipping” of an additional exon or exons in a way that restores the original correct reading frame, allowing functional protein production (Lu et al., 2000; Klein et al., 1992).

### Exon skipping in DMD

**Fig. 4:**
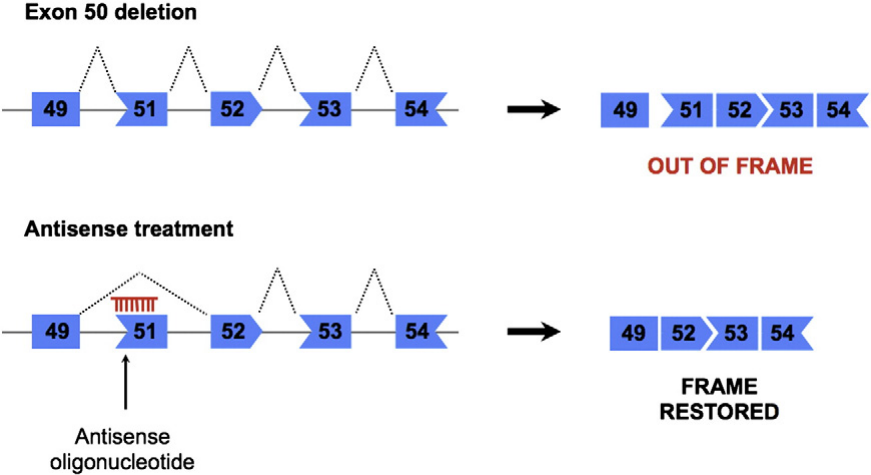
The principle of antisense-induced exon skipping in DMD. In this example exon 50 of *DMD* is deleted, causing a frameshift in the resulting spliced mRNA. Addition of an AON that recognises and hybridises to a regulatory splicing element within the sequence of exon 51, such as an ESE, mediates skipping of this additional exon by the spliceosome. This corrects the reading frame in the spliced mRNA and restores dystrophin protein production. Although the resulting mRNA lacks an additional portion of the central rod domain, this has a minimal effect on overall protein function.

As can be seen from cases of BMD, loss of a substantial part of the dystrophin central rod domain can occur with relatively little impact on protein function. The idea behind splicing therapy in DMD is therefore to convert the out-of-frame transcript into an in-frame transcript that codes for functional protein. This can be achieved by the technique of inducing exon skipping in the mutant transcript, so as to bring it back into the original reading frame (Aartsma-Rus and van Ommen, 2007). Such exon skipping can be induced using antisense oligonucleotides (AONs). These compounds are single-stranded, short lengths of nucleotides (generally not longer than 25 nt) and their sequences are designed so that they are complementary to a specific region on a pre-mRNA transcript of interest. The sequences usually target either a specific 5′ or 3′ splice site or else bind to a splicing regulatory element such as an intronic or exonic splicing enhancer (ISE or ESE) or intronic/exonic splicing silencer (ISS or ESS). Binding of an AON to the target sequence makes it unavailable to the spliceosome, interfering with the normal splicing mechanism. In this way it is possible to enhance either the inclusion or exclusion of a chosen exon from the mature mRNA (see Fig. 4). Some 70% of DMD mutations are intragenic exon deletions and are therefore potentially amenable to exon-skipping therapy (Aartsma-Rus et al., 2009a). Mutations are spread across the 79 *DMD* exons, however there are specific ‘hotspot’ regions where deletions are particularly common, such as between exons 45 and 55 where around 70% of deletions are located (Muntoni et al., 2003). AON sequences have in fact been designed for every internal DMD exon (Wilton et al., 2007). However, so far the majority of AON development has concentrated on skipping those individual exons that will benefit the greatest number of patients. Skipping exon 51, for example, can potentially be applied to 13% of all DMD mutations, exon 45 to 8.1% and exon 53 to 7.7% (Aartsma-Rus et al., 2009a). Exon skipping can also be used to treat nonsense mutations, which comprise around 15% of DMD mutations, by skipping the exon that contains the mutation itself (Spitali et al., 2009; Yokota et al., 2012). Of course, in such cases the reading frame must still be maintained and so single exon skipping for these mutations is limited to those exons that are not frame-shifting. However, this would still apply to around 47% of nonsense mutation patients.

### Double and multi-exon skipping

Targeting single exons to skip can only ever hope to treat selected groups of DMD patients with amenable exon deletions. The exon reading frame structure of the gene means that some DMD mutations (including 47% of small point and frameshift mutations) require at least 2 exons to be skipped in order to restore or maintain the reading frame (*e.g.* an exon 8 deletion requires skipping of exons 6 and 7 and a point mutation in exon 69 or 70 requires that both these exons be skipped) (Aartsma-Rus et al., 2009a). Indeed single exon skipping as a technique can treat at best up to 64% of all DMD patients. However, if it is possible to skip 2 exons using 2 separate AONs (so-called double exon skipping), the proportion of treatable patients increases by 19%, meaning that a total of 83% of all DMD patients can be potentially treated by single or double exon skipping (Aartsma-Rus et al., 2009a).

If it were possible to effect the simultaneous skipping of more than 2 exons, a greater proportion of patients could potentially be treated using a repertoire of fewer therapeutic oligonucleotide compounds. This is because the skipping of a defined set of multiple exons can potentially correct the reading frame of multiple different exon deletions. At the same time it can also be used to treat point mutations in any of the skipped exons. For example, although the majority of DMD deletions occur between exons 45 and 55, it so happens that patients with specific deletions of this region in its entirety (exons 45–55 inclusive) are known to have particularly mild BMD phenotypes (Béroud et al., 2007). This makes it an ideal multi-exon skipping target. Recently it has been shown that bodywide restoration of dystrophin expression is in fact possible through multi-exon skipping of exons 45 to 55 using a cocktail of AONs (Aoki et al., 2012). This study was done in *mdx52* mice that lack *Dmd* exon 52 and utilised 10 separate AONs intravenously. In another study, double exon skipping of exons 43–44 was shown in cultured patient myotubes using separate AONs for each exon (Aartsma-Rus et al., 2004). Unexpectedly, skipping of the seven consecutive exons 45–51 was also achieved simply by using two AONs, one for exon 45 and the other for exon 51. The fact that this appears to work suggests that splicing of exons 45–50 may occur prior to exon 44–45 splicing. This is plausible given that intron 44 is unusually long at 270 kb. A similar explanation has been suggested for the finding that AONs targeting exon 8 always lead to double skipping of exons 8–9 (Aartsma-Rus and van Ommen, 2007). In this case intron 7 is 110 kb whilst intron 8 is only 1.1 kb.

Another use of multi-exon skipping is for the potential treatment of *DMD* exon duplications. Duplications, which make up around 5–15% of mutations (Muntoni et al., 2003), present a challenge to the AON approach since discriminating the extra copy from the original is generally not feasible and skipping both copies of the exon will often lead to a frameshift. However, by skipping an additional exon or exons, the reading frame can again be restored. An exon 44 duplication was amenable to induced exon 43–44 skipping using a combination of AONs in cultured muscle cells (Aartsma-Rus et al., 2007). However, the effects of multi-exon skipping on duplications are difficult to predict and depend on which exon is in question. For example, an exon 45 duplication only required a single AON targeting exon 45. However, a larger duplication of exons 52–62 proved refractory.

### AON chemistry and design

In order to be effective therapeutic agents for the modulation of splicing, AONs ideally require a number of intrinsic properties. To start off with, the AON in question should bind in a sequence-specific manner to the target RNA transcript; the higher the specificity, the less the chance of unwanted off-target effects. Secondly, the AON should be of a chemistry that facilitates cellular uptake and activity in the appropriate intracellular compartment. Since splicing takes place in the nucleus, it is vital to design an AON that localises to the nucleus once it is taken up. In contrast, an antisense strategy seeking to utilise the siRNA pathway would best be served by an AON that remained in the cytoplasm where the processes of RNAi take place. Thirdly, because of the plethora of nucleases present *in vivo*, a well-designed AON should be resistant to nuclease degradation in order to allow it to reach its desired target intact and to maximise its potential duration of action once there. In addition to single-stranded stability, of particular importance for modified AONs is their interaction with RNAse H, which degrades RNA bound in RNA/DNA heteroduplexes. If the desired effect is transcript knockdown by degradation, the AON should be sensitive to RNAse H when bound to its target. However, for steric blocking techniques like exon skipping, the AON/RNA duplex should be resistant and not form a substrate for this enzyme. Fourthly, as with any drug, the ideal AON should have favourable pharmacokinetics and pharmacodynamics. Linked to this is of course the prerequisite that the AON should not be a toxic compound. Finally, the design of the AON must allow its effective delivery to the target tissues, whether that be a localised area such as a specific organ or brain region or body-wide systemic delivery such as to the musculature.

**Fig. 5:**
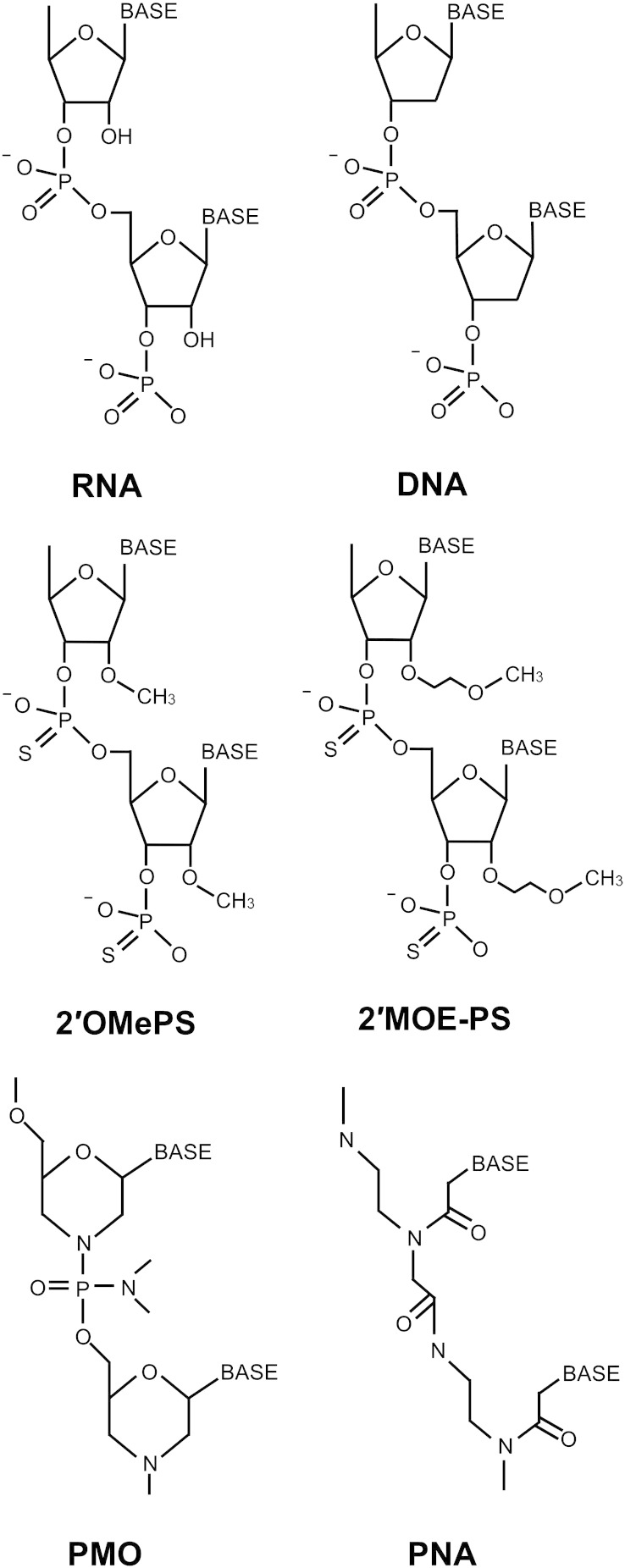
Examples of AON chemistries currently in development to treat DMD and SMA. The structures of RNA and DNA shown for comparison. 2′OMePS are based on RNA but substitute a non-bridging oxygen atom of the phosphate with a sulphur atom, making a phosphorothioate. Additionally the hydrogen of the 2′ hydroxyl group is substituted for a methyl group. 2′MOE-PS is essentially the same as 2′OMePS but instead of a methyl group has a methoxyethyl group. PMO uses a phosphorodiamidate linkage between morpholine rings instead of the normal ribose phosphate backbone. PNA also utilises an alternative backbone with peptide linkages between N-(2-aminoethyl) glycine units.

An ever-increasing range of different oligonucleotide chemistries have been developed to try to cope with these desired AON properties (Deleavey and Damha, 2012; Dias and Stein, 2002; Saleh et al., 2012). To date, several AON chemistries in particular have been utilised for splicing manipulation in DMD and SMA: 2′-O-methyl phosphorothioate (2′OMePS), 2′-O-methoxyethyl phosphorothioate (2′MOE-PS), phosphorodiamidate morpholino (PMO) and peptide nucleic acid (PNA) (see Fig. 5). Critically, in all these chemistries the ability to form Watson–Crick base-pairing with RNA is retained through the maintenance of the nitrogenous nucleobases in the correct spatial conformation. The backbone structures of these compounds, however, differ widely.

Phosphorothioates (which include 2′OMePS and 2′MOE-PS) are more closely related in structure to RNA than PMO or PNA. However, instead of utilising a phosphodiester link between nucleotides, the non-bridging oxygen atom of the phosphate group of RNA is substituted by a sulphur atom. This creates nuclease resistance and also generates chirality around the phosphorus atom, allowing formation of stereoisomers. Only the Sp diastereomer is, in fact, nuclease resistant, whilst the Rp diastereomer remains sensitive (Eckstein, 2002). Importantly, the phosphorothioate backbone modification does not, of itself, confer RNase H resistance. This is believed to be because the phosphorothioate/RNA heteroduplex adopts a conformation somewhere between B-form DNA and A-form dsRNA, which is therefore recognised by RNase H since its conformation approximates that of an RNA/DNA heteroduplex (Noy et al., 2008). Thus, resistance to RNase H instead requires 2′-O-modifications of the ribose residue, which tend to encourage a more dsRNA-like A-form conformation when bound in a heteroduplex with RNA (Deleavey and Damha, 2012). By adding a methoxyethyl group instead of a simple methyl group, 2′MOE-PS further increase nuclease resistance compared to 2′OMePS and also increase target RNA binding affinity, raising their melting temperature. Phosphorothioates also retain a negative charge. This greatly aids their solubility and means that they can be complexed easily together with cationic lipids and proteins. They also bind to plasma proteins in the circulation, which can significantly increase their half-life (Bennett and Swayze, 2010). However, they cannot be readily conjugated by covalent means to peptides. The mechanism by which they bind plasma proteins has yet to be fully elucidated but may be partly electrostatic or involve the formation of disulphide bridges with sulphur-containing amino acids.

PMOs have a six-membered morpholine ring moiety in place of ribose and the rings are joined together by phosphorodiamidate linkages. They are nuclease and RNase H resistant and have no charge on their backbone at physiological pH. They do not appear to be toxic and are very stable, the majority of compound being excreted essentially unchanged *via* the urine (Amantana and Iversen, 2005). Unlike phosphorothioates, they have no net electrical charge and therefore do not tend to interact with other non-target molecules. Although this reduces the chance of off-target effects, it also means they cannot form complexes with delivery vectors *via* electrostatic interaction. However, instead of forming non-covalent complexes, it is possible to covalently conjugate PMOs to various delivery moieties such as cell penetrating peptides (CPPs). PNAs are another AON chemistry with neutral charge, giving them similar pharmacokinetic profiles to PMOs (McMahon et al., 2002). The PNA backbone is made of repeating N-(2-aminoethyl)glycine units with nucleobases attached *via* carbonyl linkages. Like PMOs, they are resistant to RNase H and nuclease degradation and have very high target binding affinity. They can also form triplexes and even have the ability to invade double-stranded DNA (Nielsen, 2010).

Aside from AON chemistry, a separate issue is that of selecting which precise sequence to target to induce exon skipping or inclusion. At first glance, the most obvious places to target might appear to be the relevant splice sites themselves along with the intron branch point. The binding of AONs to these sequences makes them unavailable to spliceosome components, which must then instead find the next applicable splice site to use. However, by their very nature, canonical splice sites tend to have conserved consensus sequences and so targeting these sites has the potential to lead to widespread non-specific binding of AONs to other transcripts and therefore off-target effects on other genes. An alternative option that has proved effective is to target exonic splicing enhancers (ESEs). These short sequences, which are less well conserved than splice sites, bind splicing factors such as SR proteins to promote usage of a particular splice site. Their intra-exonic location means they can usually be targeted very specifically. Once again, the steric blocking of such a site on a transcript prevents the binding of relevant protein factors and this is enough to significantly alter splice site usage. For DMD, a large number of AONs targeting ESEs in many different exons have been designed and tested for exon-skipping capability *in vitro* and, in general, those AONs targeting ESEs have been shown to be more effective than those targeting splice sites (Aartsma-Rus et al., 2010a). However, choosing the exact sequence of such AONs is not entirely straightforward. The complexity of the splicing process and the considerable gaps in our knowledge about the splicing of complex genes such as *DMD* mean that the effect on splicing of a particular AON can only truly be determined experimentally. Much of AON design for DMD has thus relied on a semi-empirical approach with progression of experimental data (Aartsma-Rus et al., 2009b; Popplewell et al., 2009; Wilton et al., 2007). Defining optimal AON targets for exon 7 inclusion in SMA (see later) has in large part similarly relied on empirical methods such as minigene deletion studies of splicing (Singh et al., 2006), and AON exon walking/tiling (Hua et al., 2007). More recently, attempts have been made to formalise the process of rational AON design (Pramono et al., 2012; Aartsma-Rus, 2012). This approach uses three independent variables: co-transcriptional availability of AON binding sites within the transcript, presence of ESEs and AON target length.

### Preclinical studies of exon skipping in DMD

An early demonstration of the feasibility of the exon skipping approach was carried out in myotube cell cultures derived from DMD patients using 2′OMePS AONs transfected using polyethylenimine (PEI) to induce skipping of exon 46 (van Deutekom et al., 2001). Animal models of DMD such as the dystrophic *mdx* mouse have also been widely used. This mouse has a naturally occurring mutation in exon 23 of the *Dmd* gene that introduces a premature termination codon (Sicinski et al., 1989). *In vitro* work on *mdx*-derived myoblasts as well as *in vivo* repeated intramuscular administration demonstrated that Lipofectin-complexed 2′OMePS could be used to induce exon 23 skipping (Mann et al., 2001, 2002). Further extension of this work showed that intramuscular injection of 2′OMePS targeting the 3′ splice donor site of exon 23 restored functional levels of dystrophin in treated muscles (Lu et al., 2003). Intravenous administration of the same AON compounds resulted in widespread dystrophin restoration in skeletal muscles but not in heart (Lu et al., 2005). Subcutaneous administration of 2′OMePS AONs was found to provide more favourable pharmacokinetics and pharmacodynamics than intravenous or intraperitoneal routes (Heemskerk et al., 2010).

PMOs have also been used to induce exon skipping in DMD. Intramuscular injection of PMO targeting the same 3’ splice donor site in *mdx* exon 23 was shown to achieve local exon skipping (Gebski et al., 2003). PMO has also achieved widespread dystrophin restoration in *mdx* muscle following intravenous administration (Alter et al., 2006; Wu et al., 2010). Repeated long-term intravenous PMO administration in *mdx* mice has been shown to significantly improve muscle pathology and locomotor activity (Malerba et al., 2011). Intravenous PMO has also been tested in the dystrophic dog model, which has a point mutation at the splice acceptor site of exon 7, leading to exon exclusion, and which requires skipping of both exons 6 and 8 for dystrophin restoration. Repeated intravenous administration of a cocktail of three PMOs (two targeting exon 6 and one targeting exon 8) over 5 to 22 weeks achieved up to an average of 26% of normal dystrophin levels (Yokota et al., 2009).

One limitation of using synthetic oligonucleotides is the need for repeated dosing in order to maintain efficacy over time. One alternative is to introduce stable DNA copies of the AON sequence into the desired tissues so that therapeutic antisense sequences can continue to be expressed indefinitely. The U7 snRNA gene can be modified in this way to incorporate an AON sequence targeted to induce exon skipping in *DMD* (Goyenvalle et al., 2012). The U7 snRNP is a small ribonucleoprotein involved in histone mRNA processing. The snRNA component is 62 nucleotides long and undergoes complementary base pairing with histone pre-mRNA in order to initiate 3′ pre-mRNA processing. Modifying the central Sm protein binding site of U7 snRNA functionally inactivates it and by substituting the 5′ anti-histone sequence with an AON sequence, it is possible to induce exon skipping. When such a construct was administered within an adeno-associated virus 2 (AAV2) vector to *mdx* mice by intramuscular and intra-arterial injection, sustained production of functional dystrophin protein was observed (Goyenvalle et al., 2004). Long-term widespread dystrophin restoration was also achieved in *mdx* mice using single intravenous injection of a similar AAV1-vectored U1 snRNA construct targeted to induce skipping of exon 23 (Denti et al., 2008). However, this same kind of approach has been tested in the dystrophic dog model and whilst single local administration of AAV1 U7 snRNAs resulted in correction of dystrophic muscle phenotype and improved muscle strength, the number of dystrophin positive fibres reduced substantially over a 5-year follow-up period, suggesting that repeated administration may still be required (Vulin et al., 2012).

## Spinal muscular atrophy

As described above, the nature of the dystrophin protein and its mutated transcripts lends itself ideally to therapeutic correction through exon skipping. However, whilst this approach works well for dystrophin, the majority of human proteins are unlikely to be able to functionally tolerate the significant internal deletions of their structures generated by exon skipping. This means that only a small proportion of genetic diseases are likely to be treatable in this way. However, the process of exon skipping can be further therapeutically applied to various other pathological situations: for example to correct the aberrant usage of cryptic splice sites or, through destructive exon skipping, to downregulate the expression of a particular gene (see Aartsma-Rus et al., 2010b; van Roon-Mom and Aartsma-Rus, 2012, for reviews of this topic). Furthermore, exon skipping is not the only possible outcome of AON usage. By targeting and blocking different sequence elements such as splicing silencers, it is also possible in certain cases to use AONs to encourage inclusion of an exon where a mutation has otherwise caused it to be abnormally skipped. Perhaps the prime example of this approach can be seen in its application for the potential treatment of SMA.

SMA is an autosomal recessive neurodegenerative disorder of motor neurons. It predominantly affects the anterior horn cells (α-motor neurons) of the spinal cord and affects around 1 in 10,000 live born infants, making it the single most common genetic cause of infant mortality (Lunn and Wang, 2008; Markowitz et al., 2012; Pearn, 1978; Prior et al., 2010). The heterozygous carrier rates for mutations are around 1 in 50 in Western European populations, however carrier frequencies vary between different ethnic populations (Sugarman et al., 2012). The clinical picture can be variable and classification of SMA is divided into three main types of presentation depending on age of onset. Classical SMA type I (also known as Werdnig–Hoffman disease) presents by 6 months of age with gross motor delay, symmetrical proximal muscle weakness, generalised hypotonia and other lower motor neuron signs such as fasciculation (particularly of the tongue) and diminished or absent tendon reflexes. Such patients never achieve the ability to sit unaided and usually have poor head control. Progression tends to be rapid with increasing weakness affecting both skeletal and bulbar musculature, leading to swallowing and feeding difficulties and an increased risk of aspiration and respiratory tract infection. Worsening respiratory failure is the most common cause of mortality and death usually occurs by the age of 2 years. SMA type II typically presents slightly later, before 18 months of age. These patients can usually sit and may be able to stand but never achieve independent walking. Scoliosis tends to develop with time and respiratory muscle and bulbar weakness progressively worsens. Death is common in adolescence, although some patients may survive into their 30s. Type III SMA (Kugelberg–Welander disease) presents after 2 years with proximal muscle weakness but is a significantly milder condition. All patients are generally able to walk and few or no respiratory difficulties are encountered. Life expectancy tends to be near normal. Some more recent classifications also include type 0 (severe neonatal onset) and type IV (adult onset) but these are rarer forms.

### Molecular pathogenesis of SMA

**Fig. 6:**
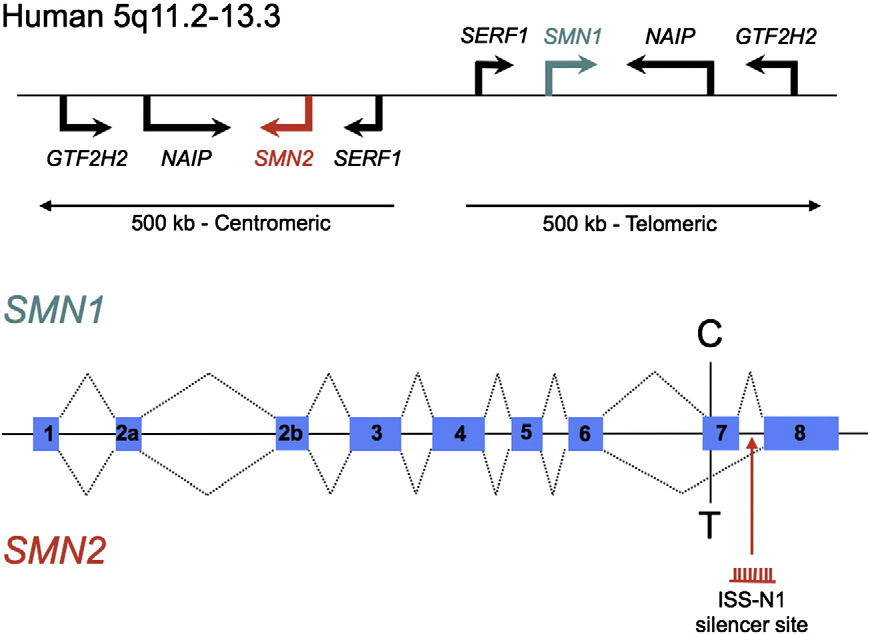
Top: The genomic arrangement of the human *SMN1* and *SMN2* locus at 5q12.2–q13.3, the result of an inverted duplication event of a 500 kb genomic region encompassing 4 genes: *SERF1*, *SMN1*, *NAIP* and *GTF2H2*. Note that the complexity of this locus means that this particular configuration may not always apply. Bottom: The normal splicing of *SMN1* joins all exons from 1 to 8 together without exclusion. A C > T transition in *SMN2* leads to exon 7 exclusion in 90% of transcripts. However, by applying an AON complementary to a splicing silencer sequence in intron 7 known as ISS-N1, inclusion of exon 7 in the mature mRNA can be restored.

SMA is caused by homozygous or compound heterozygous mutations in the *SMN1* gene which lies at chromosomal locus 5q12.2–q13.3 (Brzustowicz et al., 1990; Lefebvre et al., 1995). The genomic organisation of this locus is complex in humans because of the existence of an inverted duplication of a 500 kb long region of DNA on the long arm of chromosome 5 (see Fig. 6). This rearrangement is known to have arisen some 5 million years ago at some point prior to the divergence of human and chimpanzee evolution (Rochette et al., 2001). Mice therefore have only a single *Smn* gene, whilst higher primates such as chimpanzees generally have multiple copies. However, in humans the second copy of *SMN* has undergone slight mutation, such that its sequence differs by several nucleotides from the original. 5 single base changes were originally described (Lefebvre et al., 1995), however subsequent work demonstrated that a number of further polymorphisms can also be present (Monani et al., 1999). This human-specific version is known as *SMN2*, whilst the original is *SMN1*. *SMN2* is almost identical to *SMN1* and only one of the nucleotide alterations lies within the coding region (Lefebvre et al., 1995). It should be noted that the true nature of the SMA locus is likely to be more complex still, since it is not clear that the inverted repeat configuration always applies and indeed gene conversion events between *SMN1* and *SMN2* have been described, indicating substantial variability in this region (Burghes, 1997; Campbell et al., 1997). Both *SMN1* and *SMN2* are formed of 9 exons. However, exon 2 is itself split into two parts; exons 2a and 2b, and exon 8 is untranslated. Exon 7 is therefore the final coding exon of the gene. The mRNA is some 1.7 kb long and encodes the survival motor neuron protein, which is 38 kDa in size, 294 amino acids long and is ubiquitously expressed in all tissues.

### Splicing biology of *SMN1* and *SMN2*

**Fig. 7:**
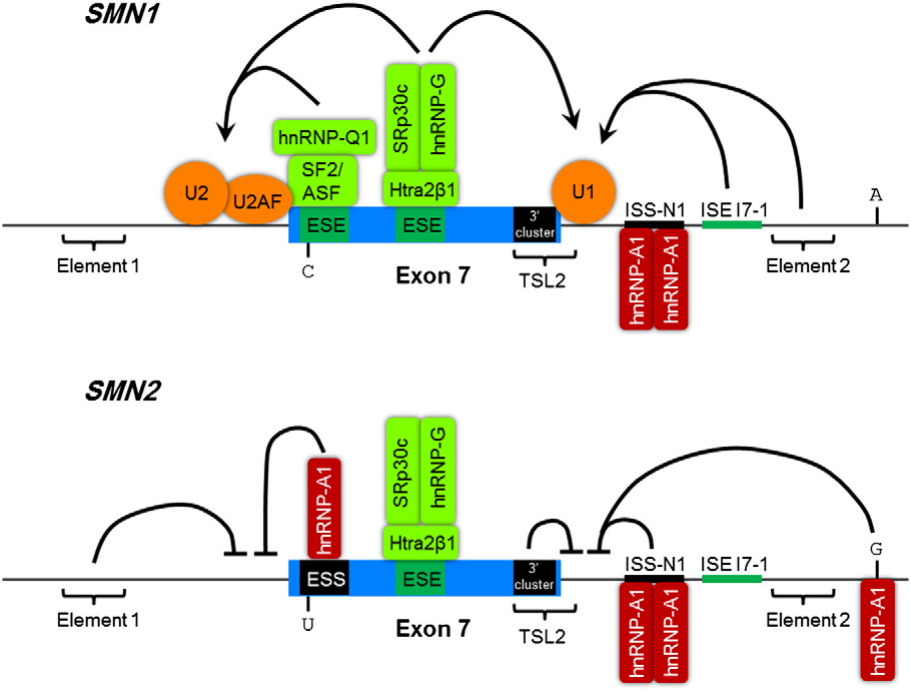
Some of the regulatory factors governing exon 7 splicing in *SMN1* and *SMN2* pre-mRNA. Effective splice inclusion of exon 7 requires the recruitment of U1 snRNP to the 5′ splice site of intron 7 and U2 snRNP to the 3′ splice site of intron 6, along with its auxiliary factor U2AF. In *SMN1*, recruitment is facilitated by an ESE near the 5′ end of exon 7 that binds splicing factor SF2/ASF, which may also act in conjunction with hnRNP-Q1 (Chen et al., 2008). An additional ESE in the middle of exon 7 enhances inclusion through recruitment of Htra2β1, which itself binds SRp30c and hnRNP-G (Young et al., 2002; Hofmann and Wirth, 2002). Two further sequence elements in intron 7 enhance exon 7 inclusion: ISE I7-1 and element 2, which is thought to form a hairpin loop (Gladman and Chandler, 2009; Miyajima et al., 2002; Miyaso et al., 2003). In *SMN2*, the C > T transition (C > U in RNA) at the sixth nucleotide of exon 7 effectively converts the ESE into and ESS, which binds inhibitory splicing factors such as hnRNP-A1 and Sam68. An upstream sequence in intron 6 known as element 1 also inhibits exon 7 inclusion, as does the ISS-N1 sequence in intron 7, which binds hnRNP-A1. The 3′ end of exon 7 harbours two inhibitory elements: the 3′ cluster and terminal stem-loop 2 (TSL2), which overlaps the 5′ splice site of exon 7 (Singh et al., 2004, 2007). Another inhibitory element is the A > G transition at the hundredth nucleotide of intron 7 in *SMN2*, which leads to hnRNP-A1 binding (Kashima et al., 2007a).

The coding mutation in *SMN2* is a C > T transition at the sixth nucleotide of exon 7 (Monani et al., 1999). This mutation itself is synonymous and so no amino acid change takes place. However, the effect of this sequence change is to destroy an ESE site, which would usually bind the splicing factor SF2/ASF, a splice-promoting SR protein (Cartegni and Krainer, 2002). Simultaneously, the new sequence acts as a new ESS by promoting binding of the heterogenous ribonucleoprotein hnRNP A1, which negatively regulates splice site usage (Kashima and Manley, 2003). The relative contributions of these two opposing mechanisms has been debated, however it has been suggested that hnRNP A1 may antagonise SF2/ASF binding (Cartegni et al., 2006; Kashima et al., 2007b). Further studies have in fact identified additional intronic sequences in introns 6 and 7 that appear to bind hnRNP A1/A2 and contribute to exon 7 exclusion in *SMN2* (Hua et al., 2008). Of particular importance is the silencing sequence ISS-N1 in intron 7. This regulatory element was initially identified by studying the effects on splicing of small deletions created in an *SMN2* minigene (Singh et al., 2006). A combination of mutagenesis analysis and RNA-affinity chromatography subsequently demonstrated that ISS-N1 contains two hnRNP A1 binding motifs (Hua et al., 2008). This inhibitory element is the major target of many AON-based treatment strategies (Hua et al., 2008; Singh et al., 2006). Other splicing factors such as Htra2β1 and Sam68 have also been implicated in exon 7 alternative splicing (see Fig. 7) (Hofmann et al., 2000; Pedrotti et al., 2010). More detailed discussions of *SMN* splicing regulation can be found in Bebee et al., 2010, and Singh and Singh, 2011.

In any case, this single C > T nucleotide change in *SMN2* is enough to cause skipping of exon 7, leading to the truncated protein variant SMNΔ7. Exon 7 codes for the C-terminal of the protein, which is important for protein oligomerisation and stability (Lorson et al., 1998). This means that SMNΔ7 is rapidly degraded (Lorson and Androphy, 2000). However, the truncated variant appears to retain a small amount of residual function and it has been shown that transgenic mice expressing SMNΔ7 in the presence of low levels of full-length SMN have a milder phenotype than those without it (Le et al., 2005). Whilst the main product of *SMN2* is SMNΔ7, a small proportion (around 10%) of *SMN2* transcript still undergoes normal splicing and so a small amount of normal SMN protein is still produced (Lefebvre et al., 1995). Since patients with SMA have no *SMN1* but usually have at least one copy of *SMN2*, enough SMN protein is present to allow developmental viability. In contrast, mice (which only have a single *Smn* gene) suffer embryonic lethality if *Smn* is knocked out. *SMN2* is therefore partially able to compensate for loss of *SMN1*, though only with reduced efficacy. Within most human populations there are variants of the *SMN* locus where secondary duplications have taken place, resulting in two, three, four or even more copies of *SMN2*. Each copy of *SMN2* is able to contribute cumulatively to the total amount of normal SMN protein produced and there is a known correlation between levels of functional SMN protein and disease severity (Lefebvre et al., 1997). This means that in general, the greater the number of *SMN2* copies a patient has, the milder the clinical phenotype of the disease (McAndrew et al., 1997). Patients with three or more copies of *SMN2* are known to have mild disease (Mailman et al., 2002). A number of other SMN isoforms have been described, including variants lacking exon 5 (SMNΔ5), exons 5 and 7 together (SMNΔ5 + 7) and a so-called axonal-SMN (a-SMN) which retains intron 3 (Gennarelli et al., 1995; Setola et al., 2007). However, the relevance of these isoforms to SMA pathogenesis is unclear.

### SMN protein

There are multiple functions ascribed to SMN protein, although the details in many cases remain quite unclear (Eggert et al., 2006). Amongst its proposed roles, it is needed for small nuclear ribonucleoprotein (snRNP) assembly, for snRNP recycling in the nucleus and it may also be involved in the cytoplasmic transport of mRNAs. snRNPs are essential components of the pre-mRNA splicing apparatus that help mediate the mechanics of the splicing reaction. They comprise various bound protein factors, together with short lengths of uridine-rich RNA (snRNAs), which have specific secondary structural conformations (Elliott and Ladomery, 2011). snRNAs are transcribed by RNA polymerase II and thus acquire a 7-methyl guanosine cap. This acts as a signal to export the snRNAs from the nucleus to the cytoplasm (except for U6 snRNA, which remains localised to the nucleus). In the cytoplasm, various maturation processes take place. SMN protein, complexed together with proteins called Gemins, directs the addition of a heteroheptamer ring of seven different Sm proteins to each snRNA (Cauchi, 2010). Sm proteins (also known as Smith-class antigens) act as a scaffold to ensure that the snRNA takes on and maintains the correct folded structure (Khusial, 2005; Tan and Kunkel, 1966). Further processing involves trimming of the 3′ end of the snRNA and hypermethylation of the 7-methyl guanosine cap to 2,2,7-trimethyl guanosine. The snRNPs are then reimported to the nucleus where they are trafficked to Cajal bodies to undergo final maturation processing.

The SMN protein complex is in fact also found colocalised with Cajal bodies themselves in structures called gems (gemini of coiled bodies), where it may play a role in snRNP recycling (Liu and Dreyfuss, 1996). Thus, SMN is vital to the cellular turnover of snRNPs and without it splicing is likely to be impaired. However, the pathogenesis of SMA does not appear to be simply down to a global dysregulation of splicing. An exon-array study of alternative splicing in the spinal cord of SMNΔ7 mice, which are null for *Smn* but express human *SMN2* and SMNΔ7 and live for up to 14 days, showed that the majority of splicing changes only occurred late in the disease process (Bäumer et al., 2009). Thus, the precise pathogenic mechanism of SMA remains somewhat unclear. In particular, the reason why there should be a predilection of the disease for a specific subset of motor neurons, with relative sparing of other tissues, remains to be understood. Studies in mouse models of SMA have shown there are cell- and tissue-specific perturbations in snRNP repertoire and it may be that the pattern of specific splicing abnormalities in motor neurons is sufficient to cause cell death (Zhang et al., 2008; Gabanella et al., 2007). Interestingly, it has recently been reported that motor neurons express significantly lower levels of functional SMN protein from *SMN2* compared to other spinal cord cells (Ruggiu et al., 2012). This appears to be due to some kind of intrinsic inefficiency of motor neurons to splice exon 7, which leads to a negative feedback mechanism that affects *SMN2* splicing itself. This may help explain motor neurons' selective vulnerability in SMA.

It has also been suggested that the pool of snRNPs involved in the minor spliceosome (a complex which facilitates the splicing of rare introns that often have the sequences AT/AC at their splice sites rather than the usual GT/AG) is more significantly affected by low SMN levels owing to the *a priori* lower expression levels of its constituent snRNAs and proteins (Coady and Lorson, 2011). Studies of SMA patient-derived lymphoblasts have indeed demonstrated low levels of minor spliceosome snRNPs and altered splicing of minor introns (Boulisfane et al., 2011). Although such AT/AC introns (also known as U12-dependent introns) are estimated to account for only around 0.34% of all vertebrate introns, they are found in higher densities in genes coding for voltage-gated ion channels, many of which are expressed in neuronal and muscular tissues (Levine and Durbin, 2001; Wu and Krainer, 1999). In this way, impaired function of the minor splicing pathway could conceivably account for the selective neuromuscular dysfunction in SMA. However, this hypothesis remains uncertain. Indeed, in a *Drosophila* model of SMA, no defects of minor class intron splicing were found, despite decreased levels of minor class snRNAs. In addition, restoring low levels of SMN expression rescued larval lethality and locomotion defects without restoring snRNA levels (Praveen et al., 2012).

Another possible explanation may relate to SMN’s function in the cytoplasmic transport of mRNAs (Rossoll and Bassell, 2009). Lower motor neurons are generally extremely long cells and the axons of those supplying the lower limbs may reach over 1 m in length. mRNAs are transported from the nucleus along axons towards the synaptic terminal for translation. In so doing, synaptic plasticity can be achieved in a more responsive and dynamic way following various stimuli (Lin and Holt, 2008). SMN has been found localised to granules within primary motor neuron growth cones that contain ribonucleoproteins (Zhang et al., 2006). However, the interpretation of these findings remains somewhat uncertain, since only a limited proportion of endogenous SMN protein was seen to colocalise with its Gemin binding partners within axons. An interaction between SMN and hnRNP R and hnRNP Q has been shown (Rossoll et al., 2002). These interact with β-actin mRNA and it has been demonstrated that decreased SMN leads to decreased β-actin mRNA and protein in axonal growth cones (Rossoll et al., 2003).

### Exon inclusion in SMA

**Fig. 8:**
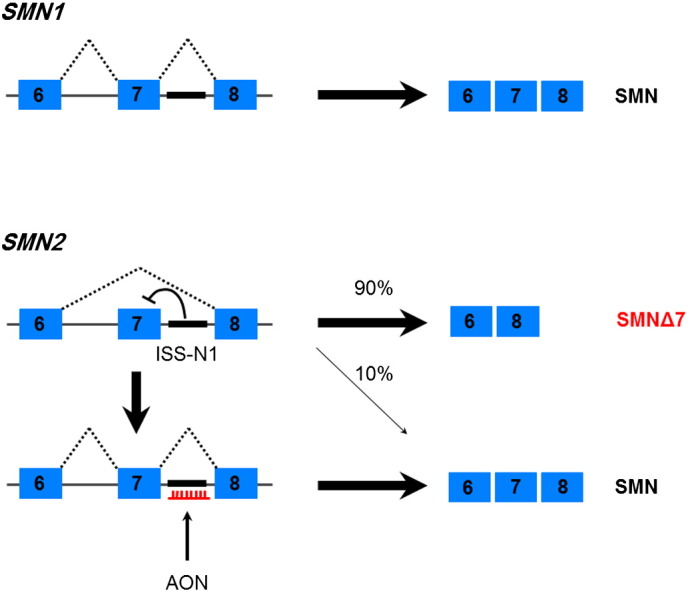
*SMN1* pre-mRNA is correctly spliced to generate full-length SMN. However, in *SMN2* skipping of exon 7 occurs in around 90% of transcripts, leading to hypofunctional SMNΔ7, whilst the other 10% remain correctly spliced. Exon 7 inclusion in *SMN2* can be induced by application of an antisense oligonucleotide (AON) targeting the inhibitory splicing element ISS-N1.

Notwithstanding the fact that the precise pathogenesis of SMA remains unclear, a valid treatment strategy for this disease is to seek to restore the expression of normal SMN protein. The presence in humans of the *SMN2* gene provides a unique opportunity through which to achieve this. One option is simply to preferentially increase the expression of *SMN2* in order to boost the total amount of transcript, about 10% of which will produce functional SMN protein. Another option is to manipulate *SMN2* splicing so as to favour retention of exon 7. A number of different strategies can be employed to increase exon 7 usage (Bebee et al., 2010). On the one hand, inhibitory splicing elements such as ISS-N1 can be antagonised, for example by masking these sequences with an AON (see Fig. 8). Alternatively, positively acting splicing factors can be recruited, for example through the use of bifunctional oligonucleotides whose sequence contains an AON but which also carries a splicing-factor binding site. The availability of alternative splice sites can be modulated *via* AON masking, and even ready-made correctly spliced transcripts can be introduced through the process of *trans*-splicing.

### Preclinical studies of exon 7 inclusion in SMA

AONs have been used to induce exon 7 inclusion by targeting inhibitory *cis*-acting sequences. This approach was shown to increase *SMN2* exon 7 inclusion in SMA fibroblasts (Hua et al., 2007). 2′MOE-PS AONs targeting the ISS-N1 sequence in *SMN2* intron 7 achieved significantly increased levels of full-length *SMN2* transcript in liver and kidney of a humanised SMA mouse model after twice weekly intravenous injections (Hua et al., 2008). These mice are hemizygous or wild type for mouse *Smn* but also have a copy of human *SMN2*. Targeting ISS-N1 with AONs has proven a potent method of promoting exon 7 inclusion; so much so that even a short 8-mer 2′OMePS AON binding to this region was still able to restore exon 7 in human SMA fibroblasts (Singh et al., 2009). A study of repeated intracerebroventricular (ICV) injection in the SMNΔ7 mouse model (*Smn*^−/−^; *SMN2*^+/+^; and *SMNΔ7*^+/+^) showed that bilateral ICV injection of newborn mice with 2′OMePS AON resulted in significant increases in SMN protein expression in brain and spinal cord at P12 (Williams et al., 2009). Body weight and motor function were also partially corrected, however the repeated ICV injection regimen caused significant mortality. Subsequently, another study by Hua et al., 2010, using ICV infusion of the same 2′OMePS chemistry targeting the same ISS site failed to show any positive effect on *SMN2* exon 7 inclusion and also detected a proinflammatory effect in brain and spinal cord. By contrast, the same study demonstrated that ICV infusion of a 2′MOE-PS chemistry led to increased exon 7 inclusion and SMN protein restoration in the central nervous system (CNS) of SMA type III mice without any proinflammatory effect (Hua et al., 2010). These mildly affected mouse models have four copies of human *SMN2* and are null for the endogenous mouse gene (*Smn*^−/−^) through knockout replacement of exon 7 with a HRPT cassette (Hsieh-Li et al., 2000). 2′MOE-PS was delivered to adult mice by micro-osmotic pump over 7 days. When applied to heterozygous (*Smn*^+/−^) animals of this same model type, spinal cord tissue demonstrated around 90% exon 7 inclusion even up to 6 months later.

PMO AONs have also been used to encourage exon 7 inclusion in SMA mouse models (Porensky et al., 2012). In this case, newborn heterozygous SMNΔ7 mice received single ICV injections of PMO targeting ISS-N1, resulting in increased full-length *SMN2* transcript, SMN protein and a prolonged increase in survival from 15 to > 100 days. This study also found that delayed ICV injection at P4 was less effective than injection at P0 and that comparing P0 combined ICV and IV administration, dual P0 and P30 ICV injection and single P0 ICV injection all resulted in similar efficacies in terms of survival (Porensky et al., 2012). Intriguingly, a study of early peripheral 2′MOE-PS AON administration (two 50 μg/g subcutaneous injections between P0 and P3) *versus* direct CNS administration *via* ICV injection (a single 20 μg dose at P1) in a severe SMA mouse model showed that peripheral systemic AON delivery was strikingly more effective than ICV injection at extending lifespan (Hua et al., 2011). These results were in spite of the finding that CNS levels of restored SMN expression were not as great after systemic administration as they were following ICV administration, suggesting an important role for peripheral SMN function in the disease process. However, the relatively increased permeability of the blood–brain barrier in neonatal mice is likely to have allowed passage of AON into brain and spinal cord and so the precise interpretation of this finding is tricky, since it is hard to judge the true dose of peripheral AON reaching the CNS in these mice and to know how comparable this is to the ICV dose. The CNS biodistribution of an AON is likely to vary greatly depending on whether it is delivered systemically or by ICV injection. It may therefore be that, even though overall SMN levels in CNS were lower in this study, enough peripherally administered AON reached the correct subset of neurons to achieve a significantly increased phenotypic effect.

A variant on the AON theme has been the development of bifunctional oligonucleotides. These compounds contain an antisense sequence but also have an additional sequence element such as a binding site for a splicing factor. 2′OMePS bifunctional oligonucleotides partially complementary to *SMN2* exon 7 and with a tail mimicking an ESE have been used to recruit SF2/ASF, thereby upregulating exon 7 inclusion in SMA patient fibroblasts (Skordis et al., 2003). Furthermore, 2′-O-methyl RNAs have been used in an SMA mouse model to upregulate full-length *SMN2* through recruitment of SR proteins for exon 7 splicing following neonatal ICV injection (Baughan et al., 2009). More recently, similar constructs of bifunctional 2′-O-methyl RNAs targeting ISS-N1 and recruiting SF2/ASF and Htra2β1 significantly increased CNS SMN protein, bodyweight and survival following ICV injection in SMNΔ7 mice (Osman et al., 2012). In a similar vein, Cartegni and Krainer (2003) reported the development of a chimeric type of compound for SMA treatment by inducing exon-specific splicing enhancement by small chimeric effectors (ESSENCE). An AON (PNA) is linked to a minimal RS domain targeted to the affected exon. The RS domain mimics the function of SR proteins to increase association of the spliceosome with the exon. This appeared to work *in vitro* but has not been tested *in vivo*.

A further extension of the AON-based strategy that parallels developments in the DMD field has been the use of anti-SMN U7 snRNA (Madocsai et al., 2005). By targeting the intron 7/exon 8 junction, an anti-SMN U7 snRNA was able to increase SMN protein levels in SMA patient fibroblasts (Madocsai et al., 2005). A similar approach has been demonstrated using modified U7 snRNAs complementary to a number of different target sequences within exon 7 and intron 7 (Marquis et al., 2007). There are a number of advantages to using snRNA-incorporated AONs. Since snRNPs are expressed in the nucleus where splicing takes place, expressing such constructs in cells automatically leads to their correct intranuclear localisation. snRNAs are also constitutively expressed at a high level, endogenously processed by cells and complexed together with proteins, enhancing their stability and half-life. A feasible strategy for long-term treatment of SMA would therefore be to introduce modified snRNA genes into motor neurons *via* a viral vector. Such an approach has been proposed using recombinant adeno-associated (rAAV) vectors, which are known to effectively transduce neurons and myocytes (Baughan et al., 2006).

An alternative type of splicing therapy can be mediated *via* the process of *trans*-splicing, whereby the mutated part of a transcript is replaced through splicing between two separate RNA molecules: one the endogenous pre-mRNA and the other an introduced correctly-spliced version of the same RNA (Mansfield et al., 2004). *Trans*-splicing can occur naturally at very low levels, however the frequency of this can be greatly increased by using an engineered *trans*-splicing RNA (tsRNA). These molecules contain a coding domain, a splicing domain featuring a 3′ splice site, polypyrimidine tract and branch point, and a binding domain, which is effectively an extended AON sequence that hybridises to the native transcript and brings the correct target region into close proximity to facilitate the *trans*-splicing event. This technique has been applied to the correction of *SMN2* splicing both *in vitro* and *in vivo*, whereby ICV injection of tsRNA molecules into SMNΔ7 mice resulted in increased CNS levels of SMN protein (Shababi and Lorson, 2012). An optimal target for the *trans*-splicing event has been identified as intron 3.

AONs are not the only compounds able to modulate splicing. A number of drugs and other small molecules have been tested for their ability to increase functional SMN protein production from *SMN2*. Valproic acid, commonly used as an anti-epileptic, has been found to activate the *SMN2* promoter (Brichta et al., 2003; Sumner et al., 2003). This is thought to occur through upregulation of the SR-like splicing factor Htra2β1 and also *via* its activity as a histone deacetylase (HDAC) inhibitor, which promotes an open chromatin conformation leading increased gene expression. Other HDAC inhibitor compounds such as phenylbutyrate (used to treat urea cycle defects), trichostatin A (an antifungal), and the anticancer agent suberoylanilide hydroxamic acid have also been shown to upregulate *SMN2* to some degree (Avila et al., 2007; Brahe et al., 2005; Hahnen et al., 2006). However, despite initial promise of such compounds along with others like hydroxyurea, riluzole and gabapentin, further trials to date have not demonstrated significant clinical improvements (Chen et al., 2010; Russman et al., 2003; Miller et al., 2001).

## Delivery of therapeutic AONs

One of the most significant and ever-present challenges facing development of any new therapeutic agent is that of drug delivery. This holds just as true for oligonucleotide-based therapies and indeed even the most efficient AON *in vitro* can only ever be as good as its *in vivo* delivery system. In DMD, the primary therapeutic target is skeletal muscle. This itself is no small task, since skeletal muscle typically makes up between 30 and 40% of total body mass (Janssen et al., 2000). Added to this, BMD patients with in-frame deletions comparable to those that would result from exon skipping treatments currently in development (*e.g.* exons 51, 53 and 45–55) have been found to express at least 40% of the dystrophin protein levels expressed by controls and so AON delivery must hope to aim for similar levels of restoration (Anthony et al., 2011). Studies using naked AONs in high enough doses have been shown to achieve effective delivery to dystrophic skeletal muscle (Wu et al., 2010). The mechanism by which muscle uptake is achieved is thought to depend on increased sarcolemmal permeability. However, the origins of this permeability are debated. One hypothesis is that since dystrophic muscle lacks dystrophin, the sarcolemmal membrane is more prone to disruption through mechanical stress and is in effect ‘leaky’ (Davies and Nowak, 2006). An alternative hypothesis invokes phospholipase A2-induced membrane permeabilisation following altered calcium channel activation (Allen and Whitehead, 2010). Either way, increased membrane permeability is thought to facilitate the passage of AONs into muscle fibres from the circulation.

In general, the polarity of charged oligonucleotides such as 2′OMePS is believed to prevent them from directly permeating cell membranes. However, more recently it has been demonstrated that such charged oligonucleotides can be directly taken up by cells in culture *via* a process called gymnosis (Stein et al., 2010). This original study utilised locked nucleic acid (LNA) phosphorothioate gapmers to achieve RNase H-mediated gene silencing in a variety of cell lines and also in mice following intravenous injection. Subsequently it has also been reported that 2′OMe phosphodiester, 2′OMePS oligonucleotides, whether flanked by LNA sequences or not, and PNAs can also be taken up by gymnosis (Torres et al., 2011). The mechanism by which gymnosis occurs is currently under investigation. However, nucleic acids are known to be internalised *via* surface glycoproteins known as scavenger receptors (Pearson et al., 1993; Saleh et al., 2006). Such receptors have a broad range of ligands including LDL, phospholipids and bacterial lipopolysaccharide. A common feature of scavenger receptor ligands is their polyanionic nature, a property shared by nucleic acids. Gymnotic uptake of negatively charged AONs may therefore also occur *via* this route. However, the extent to which gymnosis is active in the uptake of AONs *in vivo* remains to be established.

Importantly, effective AON delivery in DMD patients must by necessity also include cardiac muscle. Restoration of dystrophin in the heart is essential for successful long-term therapy, as evidenced by the fact that cardiac failure is the major cause of mortality. Indeed, restoring dystrophin in skeletal muscles alone appears to lead to increased demands on cardiac output, potentially worsening overall cardiac function (Townsend et al., 2009). Trials of naked AONs in animal models have demonstrated a consistent and significant difference in the ability of AONs to penetrate heart compared to skeletal muscle. The reasons for this lack of heart delivery are unclear and this perhaps belies the more fundamental gap in our knowledge regarding AON uptake.

### Conjugated AONs

One way of potentially improving uptake of AONs is through complexing or conjugating them with various additional carrier compounds. Such compounds can take the form of cationic lipid particles, cationic polymers, proteins and nanoparticles (Malik and Roy, 2011). In particular, much work in recent years has focussed on the use of so-called cell-penetrating peptides (CPPs) (Margus et al., 2012). These are short cationic or amphipathic peptides capable of entering cells and delivering various cargoes across the cell membrane. Early examples of naturally occurring CPPs were the Tat domain of HIV-1 and the penetratin domain of *Drosophila* antennapedia (Dupont et al., 2011). CPPs can either be noncovalently complexed to charged AONs through electrostatic interaction or else covalently conjugated to uncharged AONs. However, similar to the uptake of naked AONs, their mechanisms of cellular internalisation are not fully understood. The endocytotic pathway is generally thought to be the prevailing route of entry, although some studies have also suggested direct translocation across the cell membrane (Lundin et al., 2008; Rydström et al., 2011).

Conjugation of PMO to peptides (PPMO) and other compounds has received particular attention from the DMD exon skipping field. A dendrimeric octaguanidine PMO conjugate (Vivo-morpholino) has been tested in *mdx* mice with repeated biweekly intravenous injection resulting in expression of around 50% of normal dystrophin levels in skeletal muscle and 10% in cardiac muscle (Wu et al., 2009). Arginine-rich peptides have also been shown to significantly improve the cellular uptake and efficacy of PMOs when covalently conjugated to them (Moulton et al., 2004). One particular PPMO conjugate named B-PMO was found to restore up to 30% of dystrophin protein expression in cardiac muscle after repeated intravenous injection in *mdx* mice (Jearawiriyapaisarn et al., 2008). Intravenous B-PMO treatment was shown to improve mdx muscle pathology and strength and to prevent stress-induced cardiac failure in mice (Wu et al., 2008). Peritoneal injection of an arginine-rich PPMO was also found to be effective at restoring skeletal muscle dystrophin in *mdx* mice and also in the phenotypically more severe double knock-out (*dKO*) mouse which lacks both dystrophin and its homologue protein utrophin (Fletcher et al., 2007; Goyenvalle et al., 2010). Further refinements and modifications of the arginine-rich peptide design have led to development of the Pip (PMO-internalising peptide) series of CPPs. These peptides have two cationic arginine-rich domains either side of a hydrophobic central region and have been shown to improve both skeletal muscle and heart delivery of PMO in *mdx* mice (Yin et al., 2011). These peptides are covalently joined to the equivalent of the 5′ end of the PMO *via* a thiol-maleimide linker.

The activity of CPPs can be directed towards a particular tissue by the inclusion of a tissue-specific peptide sequence within the design. The addition to B-PMO of a 7 amino acid-long (ASSLNIA) muscle-specific peptide (MSP) found by *in vivo* phage display created the chimeric compound B-MSP-PMO, which at low doses was shown to be highly effective over B-PMO at restoring muscle dystrophin in *mdx* mice (Yin et al., 2009). Over 12 weeks, biweekly intravenous injection of B-MSP-PMO at the relatively low dose of 6 mg/kg resulted in around 50% of normal dystrophin protein levels and 100% of analysed muscle fibres showing dystrophin positivity (Yin et al., 2010).

One potential concern relating to the use of PPMOs may be toxicity at higher doses. Above a certain threshold, some arginine-rich PPMOs have caused toxic effects in animal models, including lethargy and weight loss in rats and one report of mild renal tubular damage in monkeys after repeated dosing (Amantana et al., 2007; Moulton and Moulton, 2010). Whilst the potential for such effects is an important issue for drug development, a better understanding of the toxicity mechanisms involved should in time allow appropriate chemical modifications to be made that minimise the side effect profile of these compounds.

### Brain delivery

For the treatment of SMA, a particularly significant obstacle presents itself; to cross the blood–brain barrier (BBB). The central nervous system requires a tightly regulated physiological environment in order to function correctly. To maintain this, a cellular barrier separates the tissues of the brain and spinal cord from the constituents of plasma. The main interface between CNS tissue and plasma occurs at the neurovascular unit (NVU), which comprises several components (Mäe et al., 2011). The main physical barrier is formed from the endothelial cells of brain capillaries. Surrounding this are regulatory pericytes, which regulate the integrity of the BBB and lie within the endothelial basement membrane, making contact with brain endothelial cell in an extensive network (Armulik et al., 2011). Beyond this lies a surrounding layer of astrocyte endfoot processes which envelops the entire vasculature in a layer connected by adherens junctions.

The most straightforward route for CNS administration is by direct injection. Many animal studies of AON treatment for SMA have utilised this route and intrathecal injection has been shown to achieve a therapeutically suitable CNS distribution of AON in non-human primates (Passini et al., 2011). However, direct access to the CNS remains an invasive procedure attended by concomitant procedural risks to patients. Since the treatment of neuromuscular disorders with AONs may require life-long repeated dosing, it would be highly preferable to be able to administer such agents systemically. The endothelium in the CNS lacks fenestrations and a continuous network of tight junctions prevents paracellular diffusion or transport from taking place. Any import or export to the brain must therefore take place transcellularly *via* one of several pathways. By taking advantage of these endogenous pathways such as receptor-mediated transcytosis and adsorptive transcytosis, it is possible for certain high-molecular weight drug compounds to enter the CNS (Chen and Liu, 2012). However, it remains to be seen how well AONs can be adapted for this approach. Complexing or conjugating AONs to cationic nanoparticles or cell-penetrating peptides may be able to facilitate such delivery (Hoyer and Neundorf, 2012). It may also be that extracellular vesicles in the form of exosomes, the focus of much ongoing research, will prove capable of delivering certain kinds of AON across the BBB (Lee et al., 2012).

## Clinical trials of splicing therapies in DMD and SMA

The success of AONs in animal models has led to a number of clinical trials in DMD patients. 2′OMePS AONs were the first compounds to enter such trials. Intramuscular injection of 0.8mg PRO051 (also known as GSK2402968) into the tibialis anterior muscle targeting skipping of exon 51 resulted in 64–97% of dystrophin positive fibres and 17–35% of normal dystrophin protein levels (van Deutekom et al., 2007). This led to a systemic trial of the same compound whereby 12 DMD patients were administered subcutaneous PRO051 weekly over 5 weeks at doses ranging from 0.5 mg/kg up to 6 mg/kg followed by a 12-week extension study with all patients receiving 6 mg/kg (Goemans et al., 2011). The mean terminal half-life of the drug was 29 days. Between 60 and 100% of post-treatment muscle fibres were dystrophin positive in 10 of 12 patients and the best response was expression of 15.6% of normal dystrophin protein levels. No serious drug-related adverse events were found, however mild proteinuria and increased urinary alpha-1-microglobulin levels were detected during the extension period. The study also reported an average increase of 35.2 m in patients' 6-minute walk test following treatment. Further clinical trials of GSK2402968 (now known as drisapersen) are being undertaken to compare subcutaneous 3 mg/kg *versus* 6 mg/kg dosing over 24 weeks (ClinicalTrials.gov identifier: NCT01462292). A randomised double-blind phase III trial in 180 patients is also in progress to assess clinical and functional improvement after weekly subcutaneous treatment with drisapersen at 6 mg/kg over one year (ClinicalTrials.gov identifier: NCT01254019). A recent update from Prosensa/GSK on the data from this trial (presented at the “RNA and Oligonucleotide Therapeutics” meeting at Cold Spring Harbor Laboratories, New York, 10th–13th April 2013) has reported a significant difference in patients' 6-minute walk distance after 24 weeks of treatment compared to placebo, and that a clinically meaningful difference is maintained after treatment for 48 weeks. Additional trials in progress include those looking at safety and tolerability in ambulant patients (ClinicalTrials.gov identifier: NCT01153932) and further dose-escalation studies randomised and placebo-controlled in non-ambulant patients up to 12 mg/kg (ClinicalTrials.gov identifier: NCT01128855).

PMOs have also been trialled in DMD patients. Intramuscular injections of PMO (AVI-4658) successfully induced exon skipping and dystrophin protein expression after a dose of 0.9 mg of PMO targeting exon 51 (Kinali et al., 2009). Subsequently an intravenous PMO dose-escalation study was carried out on 19 DMD patients (Cirak et al., 2011). This trial showed good tolerance of PMO (AVI-4658) from 0.5 mg/kg up to 20 mg/kg with no drug-related adverse events after weekly infusions for 12 weeks. Exon 51 skipping was detected at all doses and restored dystrophin protein was seen from 2 mg/kg upwards. Degree of response was variable but the best responses were in two patients, one of whom had 55% dystrophin positive fibres on muscle biopsy of biceps brachii post treatment, and another whose biopsy had up to 18% of normal dystrophin protein levels. The plasma half-life of the compound was between 1.6 and 3.6 h. Clinical improvement was not appreciable over this 12-week time period. However, further clinical trials are currently under way based in Ohio, USA, to assess longer term clinical outcomes following 24 weeks (ClinicalTrials.gov identifier: NCT01396239) and then 80 weeks (ClinicalTrials.gov identifier: NCT01540409) of treatment with AVI-4658 (now also known as eteplirsen). Press releases from Sarepta Therapeutics regarding progress of this trial (also presented at the 194th European Neuromuscular Centre International Workshop, Naarden, The Netherlands, 7th–9th December 2012) have reported a significant treatment benefit in terms of 6-minute walk test in patients treated with weekly intravenous eteplirsen at 30 and 50 mg/kg compared to a placebo/delayed treatment cohort at 62 weeks and beyond. Whilst these results appear encouraging, it should be noted that the patient numbers in this trial remain small and no completely untreated control group is available for comparison beyond 24 weeks. Apart from exon 51 skipping, a phase I/IIa clinical trial targeting exon 53 skipping with PMO is also currently in development.

Clinical trials for AON therapies in SMA are some way behind those for DMD. However, the first clinical trials of AONs targeting *SMN2* exon 7 splicing have recently begun (ClinicalTrials.gov identifier: NCT01494701). This trial involves the intrathecal administration of an AON compound named ISIS SMNRx to assess safety, tolerability and dosing in children affected by SMA. A number of trials have also taken place to assess the effects of other non-nucleotide based drugs in SMA. A small open-label clinical trial of valproate in SMA type III/IV patients reported increase of muscle strength and function and another study demonstrated increased *SMN2* mRNA levels following valproate treatment in SMA patients and carriers, although the effect was variable (Brichta et al., 2006; Weihl et al., 2006). The results of a phase II randomised placebo-controlled trial of valproate in SMA are awaited (ClinicalTrials.gov identifier: NCT00481013).

## Conclusions

The widespread application of splice-modulating therapies in clinical practice requires that a number of important issues are addressed. The first is the issue of optimised systemic delivery and tissue-specific targeting, which have been discussed above. The ultimate preferred delivery system for AONs would be one that allowed oral administration. However, the relatively large size of AONs as compounds makes it problematic to deliver them intact and at high enough concentrations to be systemically effective through the gastrointestinal tract. At present the major focus of AON development is concentrating on more direct routes of systemic delivery such as subcutaneous and intravenous administration. However, it is likely that in the longer term, once such drugs have become established as effective therapies, more attention will be turned to the possibility of oral AONs. A further practical issue relates to the clinical provision of such therapies within a healthcare system. It is beyond the scope of this article to discuss the potential health economics of splicing therapies. However, the prospect of delivering novel personalised oligonucleotide compounds on a patient-by-patient basis is likely to be a highly complex endeavour, requiring input from multiple areas of expertise.

Once viable treatments such as AON therapies start to enter clinical use, it is likely that the way diseases such as SMA and DMD are managed will alter considerably. In both cases, early diagnosis and treatment will be important, since neither approach is designed to replace lost tissue. In particular, exon inclusion is not a strategy that can reverse neuronal cell death in SMA. Rather, the aim of such therapy is to prevent further motor neuron degeneration and in so doing halt the progression of the disease. Thus, it may well be that the most effective application of exon inclusion therapy for SMA will require pre-symptomatic preventative deployment. This would be eminently feasible in families known to be at risk of having a child with SMA. Such families are usually those in whom an affected child has already presented and couples in this position often opt for prenatal genetic diagnosis in subsequent pregnancies. It has also been suggested that population-wide carrier screening for SMA should be carried out in order to help inform reproductive choices, however this approach remains under debate (Muralidharan et al., 2011). Development of an effective preventative therapy for SMA would, however, provide a valid and strong argument for newborn genetic screening for this condition. Such screening could conceivably be added to the panel of tests already done on newborn blood-spots (Pyatt and Prior, 2006). Since the majority of *SMN1* mutations include a deletion of exon 7, a high throughput genetic screen for common mutations would be possible. Alternatively, novel biomarkers may become available that would allow early diagnosis (Crawford et al., 2012).

Disordered pre-mRNA splicing has been implicated in an increasingly large number of medical conditions (Douglas and Wood, 2011). This review has sought to explain how two of the classic monogenic disorders of neurology can for the first time be treated through the modulation of endogenous splicing. In the case of DMD, exon skipping for the most common deletions is rapidly approaching mainstream clinical application. However, the pathway for the development of essentially individualised novel therapeutic compounds for the treatment of the small numbers of patients with rarer mutations is less clear (Muntoni et al., 2010). Whilst this is less of an issue for SMA, it is highly relevant to DMD, where mutations are spread right across the gene. Furthermore, if double or multi-exon skipping is to become a clinical reality, thought needs to be given to how the use of different yet complementary AON compounds, which individually may not be of therapeutic benefit unless used in combination, can be developed and tested in a way that satisfies drug regulatory policy. It may be, for example, that a new type of streamlined clinical trial pathway is required specifically for oligonucleotide drug development; one that is somehow able to take into account both the necessity of adequate patient safety and the pressure of desperate clinical need. Whatever the solution, it is clear that the current legislative and regulatory frameworks that govern drug development were not designed to cope with sequence-specific oligonucleotide technology. If this kind of truly personalised genetic medicine is to become widely available and applicable to many patients with many different types of mutation, significant changes will have to be made to the regulatory process.
